# Necroptosis: A Novel Pathway in Neuroinflammation

**DOI:** 10.3389/fphar.2021.701564

**Published:** 2021-07-12

**Authors:** Ziyu Yu, Nan Jiang, Wenru Su, Yehong Zhuo

**Affiliations:** ^1^ State Key Laboratory of Ophthalmology, Zhongshan Ophthalmic Center, Sun Yat-sen University, Guangzhou, China; ^2^ Department of Pediatric Ophthalmology, Guangzhou Children’s Hospital and Guangzhou Women and Children’s Medical Center, Guangzhou Medical University, Guangzhou, China

**Keywords:** neuroinflammation, necroptosis, ripk1, ripk3, mlkl, necrostatin-1

## Abstract

Neuroinflammation is a complex inflammatory process in the nervous system that is expected to play a significant role in neurological diseases. Necroptosis is a kind of necrosis that triggers innate immune responses by rupturing dead cells and releasing intracellular components; it can be caused by Toll-like receptor (TLR)-3 and TLR-4 agonists, tumor necrosis factor (TNF), certain microbial infections, and T cell receptors. Necroptosis signaling is modulated by receptor-interacting protein kinase (RIPK) 1 when the activity of caspase-8 becomes compromised. Activated death receptors (DRs) cause the activation of RIPK1 and the RIPK1 kinase activity-dependent formation of an RIPK1-RIPK3-mixed lineage kinase domain-like protein (MLKL), which is complex II. RIPK3 phosphorylates MLKL, ultimately leading to necrosis through plasma membrane disruption and cell lysis. Current studies suggest that necroptosis is associated with the pathogenesis of neuroinflammatory diseases, such as Alzheimer’s disease, Parkinson’s disease, and traumatic brain injury. Inhibitors of necroptosis, such as necrostatin-1 (Nec-1) and stable variant of Nec (Nec-1s), have been proven to be effective in many neurological diseases. The purpose of this article is to illuminate the mechanism underlying necroptosis and the important role that necroptosis plays in neuroinflammatory diseases. Overall, this article shows a potential therapeutic strategy in which targeting necroptotic factors may improve the pathological changes and clinical symptoms of neuroinflammatory disorders.

## Background

Inflammation, which is usually caused by injury or infection (Andersson and Tracey, 2011), are fundamentally distinguished by pathology (Kearney and Martin, 2017) between acute and chronic forms of inflammation. Acute inflammation responds to irritants in the early stages, which is an essential response that prepares the body to repair damaged areas during acute inflammation and neuroinflammation, including traumatic brain injury (TBI), stroke, and encephalitis (Huang et al., 2018a; Zhang et al., 2018; Venkatesan et al., 2019). Chronic inflammation is caused by persistent stimuli, which leads to injury of the nerve tissue, resulting in neurodegeneration and inducing neuroinflammation into a vicious cycle (Nasef et al., 2017), including Alzheimer’s disease (AD), Parkinson’s disease (PD), and amyotrophic lateral sclerosis (ALS) (Dong et al., 2018; Ren et al., 2018; Lee et al., 2019). A variety of immunocytes are involved in inflammation (Mack, 2018; Tsai et al., 2018). Acute inflammation is dominated by neutrophil infiltration (Pinho-Ribeiro et al., 2018; Kapur et al., 2019), whereas chronic inflammation is often accompanied by macrophage and lymphocyte infiltration (Cao et al., 2019; Faissner et al., 2019; Lodygin et al., 2019; Na et al., 2019).

Neuroinflammation is a complex inflammatory response of the nervous system that may be triggered by various pathogens or toxins and induce immunocyte infiltration and activation (Stephenson et al., 2018). Eventually, the effects lead to neuronal and/or axonal degeneration or death (Estes and McAllister, 2016). A feature essential to maintain neuroplasticity is neuroinflammatory homeostasis. Homeostasis is regulated by the interaction between neurons, glial cells and vascular endothelial cells. Homeostatic imbalance caused by different reasons (such as injury, infection or stress) may have similar pathological manifestations (DiSabato et al., 2016).

However, the mechanisms of neuroinflammation remain unclear in different situations. To better understand the inflammatory disease neuritis, acute and chronic neuroinflammation will be discussed separately in the following sections.

## Necroptosis

Historically, two forms of cell death, apoptosis and necrosis, have been recognized because of their important roles in homeostasis, development, and pathogenesis (Pasparakis and Vandenabeele, 2015). In the past, necrosis was thought to be accidental death due to excessive cytotoxic damage, carried out *via* conventional molecular events (Vanden Berghe et al., 2014). In contrast, apoptosis is defined as programmed cell death. Under a microscope, apoptosis is characterized by apoptotic bodies, nuclear pyknosis and fragmentation, and an intact cell membrane (Kerr et al., 1972; Choo et al., 2019). However, an increasing number of studies have described another form of necrosis that performs as programmed and regulated cell death, named necroptosis.

Morphologically, necroptosis has the following characteristics: (1) it resembles necrosis–dying cells cluster together, with disrupted membranes, swollen cell bodies and organelles, and fragmented chromatin; (2) a large quantity of inflammasomes; (3) autophagy (Vanden et al., 2013). Compared with apoptosis, necroptotic cells passively pass through the damaged membrane into the extracellular matrix (Zhang et al., 2017a).

At the molecular level, intracellular and extracellular stimuli and corresponding ligands of the death receptor family trigger necrosis (Zhou and Yuan, 2014). The currently known key components of the necroptotic signaling pathway are receptor-interacting protein kinase 3 (RIPK3) and its substrate, mixed lineage kinase domain-like protein (MLKL), which is a pseudokinase (Yuan et al., 2019). Moreover, small molecules, such as necrostatin-1 (Nec-1), Nec-5, and Nec-7, are thought to inhibit the necroptotic signaling pathway (Gonzalez-Juarbe et al., 2015; Strilic et al., 2016; Fuji et al., 2018). Nec-1 is an ATP-competitive allosteric inhibitor of RIPK1. In a mouse stroke model, Nec-1 was able to reduce brain damage caused by reperfusion (Chu et al., 2018). MicroRNA-155 is also considered an inhibitor of RIPK1 (Liu et al., 2011).

Necroptosis is involved in many pathological processes, such as trauma, cerebral ischemia-reperfusion injury, and inflammatory diseases (Wang et al., 2012; Li et al., 2019; Ni et al., 2019; Zhang et al., 2019). Necroptosis has been reported to play a crucial role in the pathogenesis of certain neuroinflammatory disorders (Ito et al., 2016; Chia et al., 2018; Zhang et al., 2019). Because necroptosis shows the potential to be a target for intervention in neuroinflammatory disorders, it has garnered increasing focus from researchers. In our review, the molecular mechanisms of necroptosis and the role of necroptosis in the development and progression of acute and chronic neuroinflammatory disorders will be described, which may be helpful for finding treatments for neuroinflammatory diseases.

## Necroptosis Pathways

### Mechanisms Regulating TNFR1-Induced Necroptosis

**FIGURE 1:**
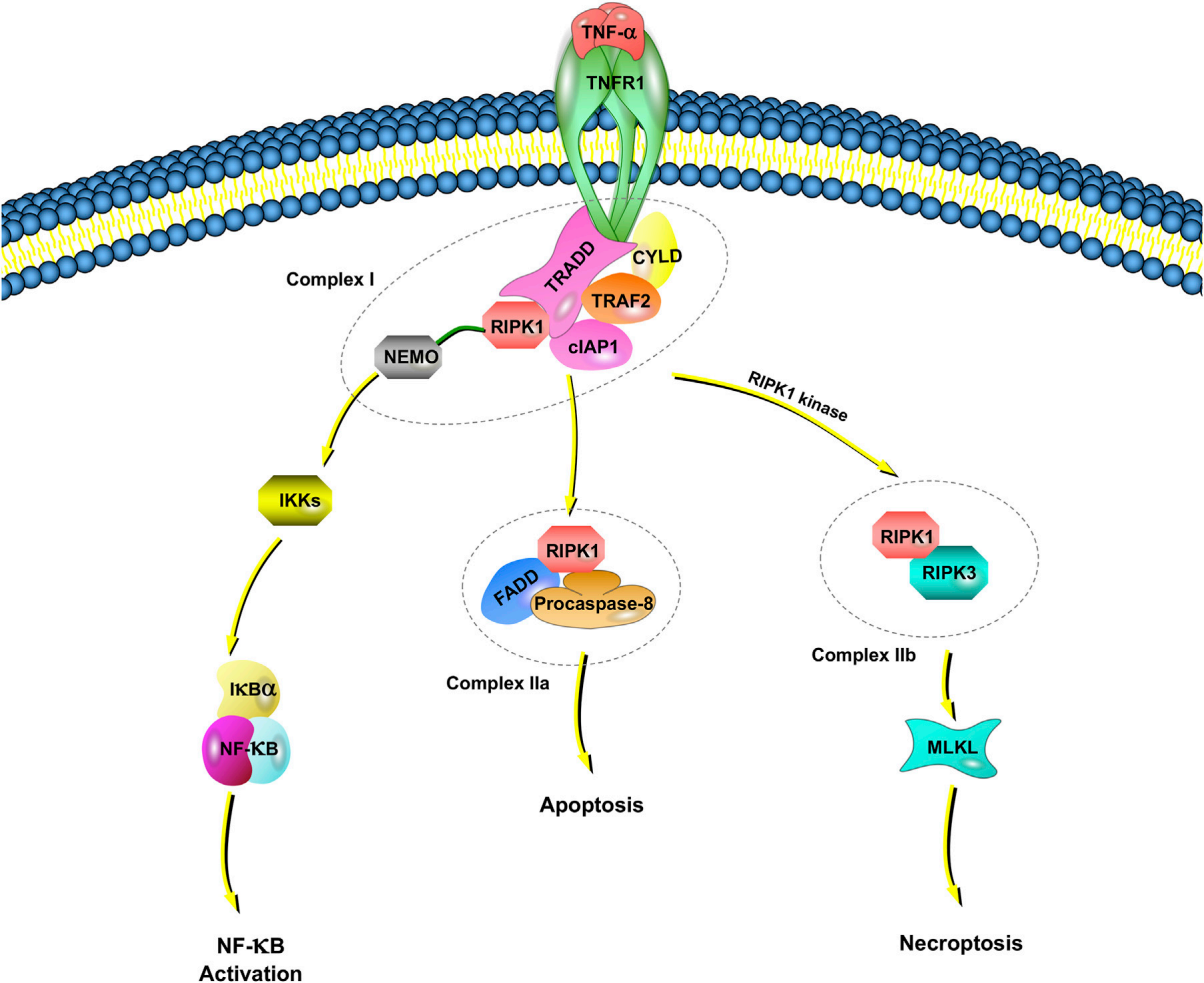
TNFR1-dependent necroptosis pathways. TNF-⍺ binds to TNFR1 and recruits a series of proteins, including TRADD, RIPK1, TRAF2, cIAP1, CYLD, and NEMO, which is called complex I. NEMO recruits IKK⍺/IKKβ, resulting in the IKK-mediated phosphorylation of IkB⍺. Once IkB⍺ is phosphorylated, NF-kB signaling pathways are activated. When NF-κB activation is inhibited, deubiquitinated RIPK1, FADD and procaspase-8 are assembled as complex IIa. Complex IIa is involved in apoptosis by activated caspase-8 and cleavage RIPK1. When there is a lack of caspase-8, the cIAP1 in complex I rapidly ubiquitinates RIPK1, leading to the combination of RIPK1 and RIPK3, which is called complex IIb. Complex IIb leads to MLKL-mediated necroptosis.

Tumor necrosis factor receptor 1 (TNFR1), which has been reported by studies of TNF signaling in necroptosis, induces the expression of many genes that regulate inflammation (Liu et al., 2014). However, under some conditions, TNF-⍺ also induces cell death (Annibaldi and Meier, 2018; Lafont et al., 2018). TNF-induced cell death requires TNF-⍺ to bind to TNFR1 on the cell membrane and recruit a series of proteins in the cell to form different complexes (Micheau and Tschopp, 2003; Amin et al., 2018). Among them, complex I includes TNFR-associated death domain (TRADD), RIPK1, TNFR-associated factor 2 (TRAF2), the cellular inhibitor of apoptosis protein 1 (cIAP1), cylindromatosis, and the ubiquitin complex (Amin et al., 2018) (Figure 1).

An I_k_B kinase (IKK) complex consisting of a subunit essential regulator (nuclear factor-kappa B essential modulator, NEMO, also named IKKγ) and two catalytic subunits (IKK⍺ and IKKβ) plays an important role in mediating immunoinflammatory responses and promoting cell survival and oncogenesis (Silke and Brink, 2010). NEMO recruits IKK⍺/IKK*β*, resulting in the rapid and selective IKK-mediated phosphorylation of I_k_B⍺. I_k_B⍺ activates NF-_k_B and upregulates genes encoding prosurvival and proinflammatory molecules.

In complex I, E3 ligase (e.g., cIAP1) rapidly ubiquitinates RIPK1. The ubiquitination of RIPK1 is important for regulating its kinase activity. Inhibiting RIPK1 ubiquitination by antagonizing E3 ligase leads to an increased sensitivity of cells to TNF-induced necroptosis (Feoktistova et al., 2011). When NF-κB activation is inhibited, deubiquitinated RIPK1, Fas-associated death domain (FADD) and procaspase-8 are assembled into the death-inducing signaling complex, and the complex finally dissociates from the plasma membrane, now referred to as Complex IIa. Complex IIa is involved in apoptosis by affecting the activation of caspase-8 and the subsequent cleavage of RIPK1 (Lin et al., 1999). When RIPK1 is deubiquitinated, the formation of complex IIb, which is also called the necrosome, is facilitated by RIPK1, RIPK3, MLKL, FADD and procaspase-8.

Recently, cellular FADD-like IL-1β-converting enzyme-inhibitory protein (c-FLIP), a noncatalytic inactive homolog of caspase-8, was reported to take part in the regulation of necroptosis (Tsuchiya et al., 2015). If c-FLIP long is involved in the composition of the heterodimer, caspase-8 maintains high proteolytic activity to inhibit the association of RIPK1, RIPK3 and FADD, thus suppressing necroptosis (Tsuchiya et al., 2015). However, caspase-8 has no proteolytic activity if it is formed by c-FLIP short; RIPK1 and RIPK3 can therefore be assembled to promote necroptosis. Inhibitors of the necroptotic signaling pathway, such as Nec-1, Nec-1s (a stable variant of Nec-1) and other small molecules, have been widely applied to elucidate the molecular mechanisms of necroptosis (Takahashi et al., 2012). Necroptosis is also regulated by the pseudokinase MLKL, which is a functional RIP3 substrate. The subsequent conformational change in MLKL causes rapid cell membrane breakage (a morphological sign of necrosis) by the formation of disulfide bond-dependent amyloid-like polymers (Wang et al., 2014).

### Necroptosis Regulation by Toll-like Receptors

**FIGURE 2:**
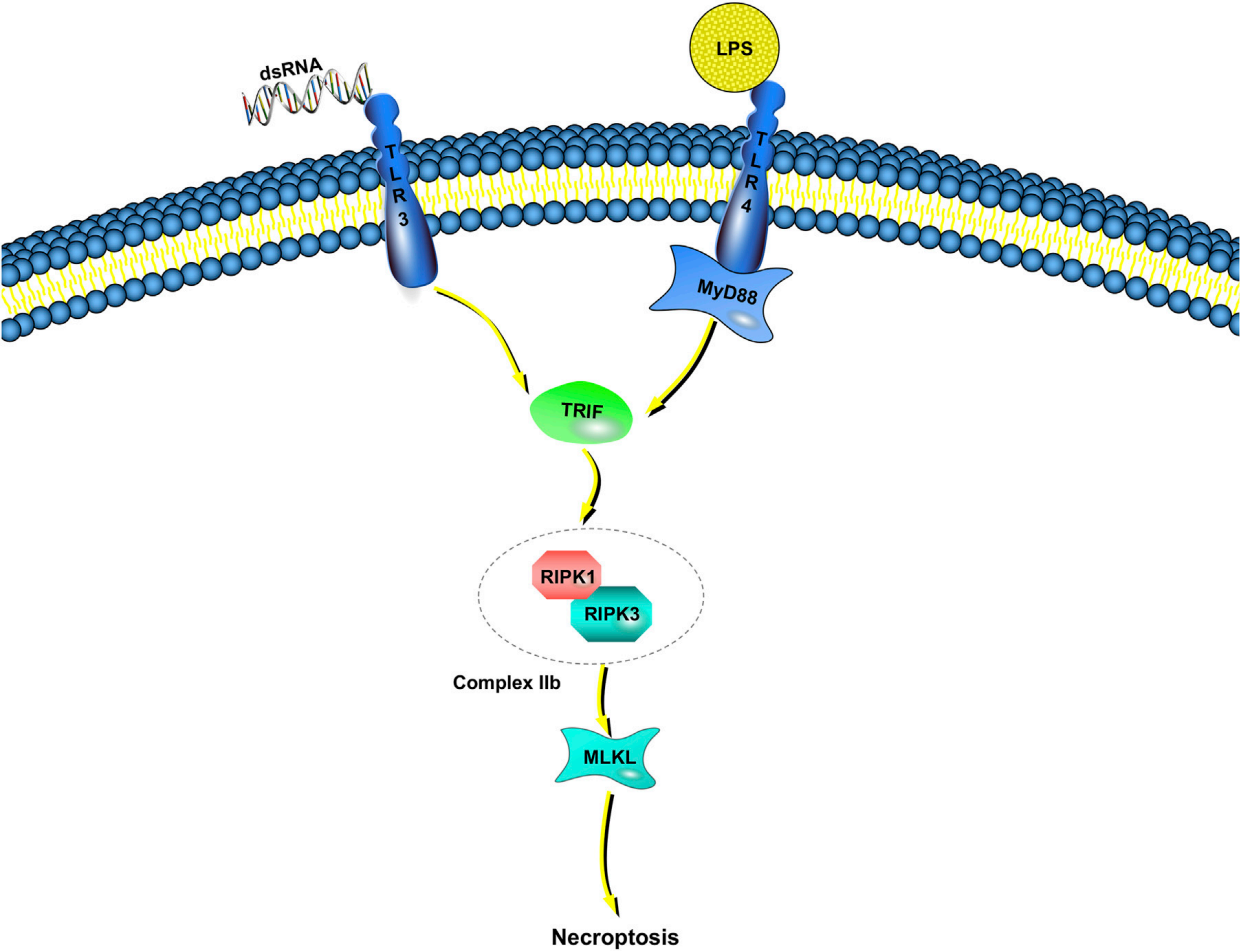
TLR-dependent necroptosis pathways. Engagement of TLR3/TLR4 with dsDNA or LPS induces the interaction between TRIF and complex IIb, which is combined RIPK1 with RIPK3. If caspase-8 catalytic activity is impaired, complex IIb triggers MLKL-dependent necroptosis. TRIF is the only adaptor protein of TLR3, whereas the TLR4 pathway can be activated by either TRIF or myeloid MyD88.

Unlike TNF-induced necroptosis, the molecular mechanisms of microbial-triggered necroptosis are more elusive (Figure 2). Innate immunocytes and macrophages, for example, detect microbial activities and initiate antimicrobial responses though pattern-recognition receptors (Kawai and Akira, 2010). Some of the most well-characterized members of the pattern-recognition receptor family are the TLRs (Fitzgerald and Kagan, 2020). TLRs respond to many pathogen-associated molecular patterns (bacteria, viruses, fungi, parasites, etc.) (Tartey and Takeuchi, 2017). TLR3 detects viral double-stranded RNA or artificial analogs, whereas TLR4 responds to lipopolysaccharide (LPS) (Alexopoulou et al., 2001; Fitzgerald et al., 2003). Toll/IL-1 receptor domain-containing adaptors are recruited after the binding of TLR3 and TLR4 to their corresponding ligands (Netea et al., 2009). Then, inflammatory cytokines are released, leading to type I interferon (IFN) responses. There is only one adaptor protein for TLR3, Toll/IL-1 receptor domain-containing adaptor inducing IFN-β/TIR domain-containing adaptor molecule 1 (TRIF/TICAM-1), whereas the TLR4 pathway can be activated by either TRIF or myeloid differentiation factor 88 (MyD88) (Fitzgerald et al., 2001; Akira and Hoshino, 2003; Kagan et al., 2008).

In addition to these immune responses, LPS induces necroptosis instead of apoptosis in human macrophages when inhibitors (such as zVAD-fmk) suppress the activation of caspase-8 (Lawlor et al., 2015). Poly (I:C) leads to Jurkat cell (human leukemia T cell) apoptosis when combined with IFN-γ, but the absence of caspase-8 or FADD results in necroptosis rather than apoptosis (Alvarez-Diaz et al., 2016; Boyd-Tressler et al., 2017). Although these results show that necroptosis may be achieved through TLR3 and TLR4 pathways, how to proceed is still unclear (He et al., 2011).

## Necroptosis in Acute Neuroinflammation

### Traumatic Brain Injury

TBI is a disease caused by external forces or other pathological changes in the brain, accompanied by changes in brain function (Maas et al., 2008; McDonald et al., 2016). The mortality rate of severe TBI is high and is estimated at 30–40% in unselected populations in observational studies (Rosenfeld et al., 2012). Some TBI patients lose their lives, and even survivors suffer from enormous physical, mental, emotional and cognitive impairments that undermine the lives of patients and their families and cause enormous losses to society (Limb, 2014; Jenkins et al., 2016).

**TABLE 1:** Necroptosis in acute neurodegenerative diseases.

Disease	Regulatory factors	Synthetic inhibitors	Comment	Reference
Traumatic brain injury	RIPK1	Nec-1	Hypothermia inhibited necroptosis pathway though down-regulation of RIPK1, in moderate TBI models of rats.	The, (2018)
			Necrostatin-1 inhibited apoptosis and autophagy simultaneously.	Wang et al. (2012)
	RIPK3		Oxidative stress, inflammation and apoptosis in astrocytes, which dependent on AMPKa activation, were attenuated by RIPK3-ablation.	Lakhan et al. (2009)
			RIPK3-knockout (KO) attenuated cognitive dysfunction and activation of glia cells in TBI injuryed mice	Lakhan et al. (2009)
	MLKL		RIPK1, MLKL and pro-inflammation cytokines increased in rat FPI models.	Zhang et al. (2017b)
Stroke	NLRP3		NLRP3 inflammasome was found in both immune cells and necroptotic neuron when caspase is inhibited by Q-VD-OPH	Bai et al. (2019)
	RIPK1	Nec-1	Pretreatment with Necrostatin-1 ameliorated cell death by reducing the interact of increased RIPK3 with RIPK1.	Teng et al. (2018)
	RIPK3		Expression level of RIPK3 was increased after ICH.	Teng et al. (2018)
Encephalitis	MLKL		In mice model, the expression of MLKL in neurons was upregulated when JEV infected, while deletion of MLKL mitigated the progression of JE and down-regulated the level of inflammatory factors.	Bian et al. (2017)
	RIPK3		RIPK3 restricts WNV pathogenesis by inhibiting necroptosis in a mouse WNV encephalitis.	Barnett, (2019)
			RIPK3-/- mice was more likely to survive compared to wild-type controls, while lacking the necroptotic effectors (such as MLKL, or both MLKL and caspase-8)	Barnett, (2019)

FBI, Fluid precussion injury; ICH, Intracerebral hemorrhage; JEV, Japanese encephalitis virus; MLKL, Mixed lineage kinase domain-like protein; Nec-1, Necrostatin-1; NLRP3, NLR Family Pyrin Domain Containing 3; RIPK1, Receptor-interacting protein kinase 1; RIPK3, Receptor-interacting protein kinase 3; TBI, Traumatic brain injury; WNV, West Nile Virus.

**TABLE 2:** Necroptosis in chronic neurodegenerative diseases.

Disease	Regulatory factors	Synthetic inhibitors	Comment	Reference
AD	RIPK1	Nec-1	Nec-1 reduced A and tau abnormalities in AD animal model.	Ofengeim et al. (2017)
			RIPK1-dependent transcription promoted microglia and lysosomal defects to increase accumulation of amyloid plaques	Hirsch and Hunot, (2009)
	MLKL		MLKL, which was required by necroptosis, was regulated by Flotillin and/or ALI syntenin-1 in AD.	Xu et al. (2017)
PD	RIPK1	Nec-1	Inhibiting th enzyme alleviated the progression of PD by blocking RIPK1 active	Dionísio et al. (2019)
			Nec-1 protected dopaminergic neurons against injury	Chia et al. (2018)
	RIPK3		The level of RIPK3 in the SN were increased in the autopsy of PD patients.	Wu et al. (2015)
	MLKL		The level of MLKL were found upregulated in the body of PD patients.	Wu et al. (2015)
	Parkin		The loss of parkin protected microglia cells from zVAD-induced necroptosis.	Re et al. (2014)
ALS	RIPK1	OPTN	The levels of RIPK1 were elevated in spinal cord extracts from Tg SOD1G93A	Politi and Przedborski, (2016)
			OPTN suppressed RIPK1-dependent necroptosis signaling by regulating its turnover.	Selik et al. (1984)
AIDS	Caspase-8		Upregulation of caspase-8 lead to disorder of HIV-specific CD8(+) T cell proliferation, by promoting necroptosis and cell death.	Gaiha et al. (2014)
Glaucoma and other retinopathy	RIPK1	Nec-1, Cpd27, RIC	Low-levels of RIPK1 and RIPK3 reduced microglia necroptosis when TLR4 def and suppressed retinal inflammation.	Cruz et al. (2018)
			Nec-1, Cpd27 and RIC inhibited downstream pathways following RIPK1 activate including necrosome composition and mitochondrial dysfunctio.	Qin et al. (2018)
	RIPK3		RIPK1-and RIPK3-dependent necroptosis existed in microglis of mice with degenerative, or acute retinal neural injury	Cruz et al. (2018)

Aβ, Amyloid-β; AD, Alzheimer’s disease; AIDS, Acquired Immune Deficiency Syndrome; ALS, Amyotrophic lateral sclerosis; HIV-1, Human immunodeficiency virus 1; MLKL, Mixed lineage kinase domain-like protein; Nec-1, Necrostatin-1; OPTN, optineurin; PD, Parkinson’s disease; RIC, RIPK1-inhibitory compound; RIPK1, Receptor-interacting protein kinase 1; RIPK3, Receptor-interacting protein kinase 3; SN, Substantia nigra; TLR, Toll-like receptor.

A series of studies in 2012 revealed that multiple cell death pathways participated in the development of TBI and that Nec-1 simultaneously inhibited apoptosis and autophagy (Wang et al., 2012). Pathological and biochemical changes related to necroptosis in a rat model of fluid percussion injury (FPI) were observed by Liu (Liu et al., 2016). In an early phase (6 h) after TBI, RIPK 1 and 3, MLKL, HMGB1 and proinflammatory factors (such as TNF-α, IL-6 and IL-18) were increased in the cortex (Liu et al., 2016). Posttraumatic hypothermia (33 °C) led to decreases in necroptosis regulators, proinflammatory cytokines and brain injury in TBI rats compared to treatment with normal temperature (You et al., 2008). Notably, by targeting necroptosis signaling after TBI, the injured central nervous system (CNS) can be protected from tissue damage and inflammation (You et al., 2008). The following year, hypothermia was reported to significantly reduce RIPK-1 upregulation in moderate TBI rat models, which may inhibit the necroptosis pathway after moderate TBI (Zhang et al., 2017b). Moreover, cognitive dysfunction and glial activation could be observed in TBI mice, and these changes were attenuated in RIPK3-knockout (KO) mice (Liu et al., 2018). Notably, *in vitro* studies have shown that RIPK3-knockdown of astrocytes can reduce oxidative stress, inflammation and apoptosis, which is dependent on the activation of adenosine 5‘-monophosphate-activated protein kinase-alpha (AMPKα) (The, 2018). In conclusion, inhibition of RIPK3 may be a therapeutic target against cerebral damage by suppressing immunoinflammatory responses, oxidative stress and apoptosis.

### Stroke

Stroke is a broad term that includes diseases caused by blockage or bleeding of blood vessels that supply the brain (Lakhan et al., 2009; The, 2018). Its incidence remains high, while the number of approved treatment methods is low (Ceulemans et al., 2010). As society ages, the number of stroke patients continues to increase and will become an important socioeconomic burden, as 80% of stroke patients remain disabled (Durukan and Tatlisumak, 2007; Candelario-Jalil, 2009).

Ischemia-reperfusion injury (IRI) is a common feature when the blood supply is restored after a period of ischemia (Wu et al., 2018). Several studies have suggested that different mechanisms, including oxidative stress, leukocyte infiltration, platelet adhesion and aggregation, blood-brain barrier disruption, complement activation, and mitochondria-mediated mechanisms, are involved in the pathogenesis of IRI in the nervous system (Bavarsad et al., 2019). In 2005, scientists demonstrated that delayed ischemic brain injury in a mouse model was due to necroptosis, a mechanism different from apoptosis (Degterev et al., 2005). This finding shows a new therapeutic target for stroke with an extended period of time for neuroprotection. The researchers also identified a specific and potent small-molecule inhibitor of necroptosis, Nec-1 (Chu et al., 2018).

Studies in 2018 indicated that necroptosis is probably involved in intracerebral hemorrhage (ICH) (Chu et al., 2018). In an *in vivo* mouse ICH model, pretreatment with Nec-1 protected astrocytes (Zille et al., 2017). Intracerebroventricular treatment with Nec-1 was helpful in reducing cell death, cerebral edema, hematoma volume and neurological score insufficiency (Su et al., 2015; Shen et al., 2017). In addition, the expression level of RIPK3 was increased after ICH, and pretreatment with Nec-1 reduced the interaction between RIPK3 and RIPK1 and promoted cell survival after ICH (Shen et al., 2017). The nucleotide-binding oligomerization domain (NOD)-like receptor (NLR) family pyrin domain-containing 3 (NLRP3) inflammasome was reported to participate in necroptosis, accompanied by changes in inflammatory factors such as IL-1β. The study authors also found that NLRP3 is expressed not only in immune cells, such as microglia, but also in necroptotic neurons when caspase is inhibited by Q-VD-OPH (Teng et al., 2018). Furthermore, in an IRI rat brain model, NLRP3 inflammasome deficiency protects cerebral tissue from injury, suggesting that the NLRP3 inflammasome plays an important role in neuronal necroptosis.

### Encephalitis

Some acute neuroinflammation is caused by viruses, such as Japanese encephalitis virus (JEV) and West Nile virus (WNV) (Turtle and Solomon, 2018; Bai et al., 2019). In Asia and the Western Pacific, the most common pathogen of viral encephalitis is JEV (Erlanger et al., 2009). JEV infection and inflammation lead to neuronal death, and subsequent cytotoxicity induces deterioration of Japanese encephalitis (JE) (Bai et al., 2019). In 2017, for the first time, necroptosis was discovered to be a reason for neuronal loss in the JE brain (Bian et al., 2017). When JEV infected neurons, the expression of MLKL was upregulated *in vitro* and *in vivo*. The loss of MLKL attenuates the progression of JE and the level of inflammatory factors in a rodent model (Bai et al., 2019).

The same year, RIPK3 was found to restrict WNV pathogenesis independently of cell death in a mouse model of WNV encephalitis (Daniels et al., 2017). Ripk3^−/−^ mice showed higher mortality rates than wild-type (WT) mice, whereas mice with a deficiency of MLKL or MLKL and caspase-8 were little affected (Daniels et al., 2017). The expression of neuronal chemokines was inhibited in Ripk3^−/−^ mice, and recruitment of T cells and other immunocytes in the CNS was reduced, which led to an enhanced susceptibility to death in Ripk3^−/−^ mice. These studies demonstrate the multiple functions of RIPK3 in the pathogenesis of viral diseases. Thus, RIPK3 may be a key regulatory factor during CNS immune processes.

## Necroptosis in Chronic Neuroinflammation

### Alzheimer’s Disease

AD is recognized by gradual memory worsening, personality disorders and a decline in general cognition (Scheltens et al., 2016; Barnett, 2019). It is the sixth leading cause of death in the United States, causing more than five million deaths (Alzheimer’s Association., 2016). Neuropathologically, the main characteristic of AD is severe neuronal loss, paraprotein [tau, amyloid-β (Aβ)] accumulation, and significant neuroinflammation (Parbo et al., 2018; Whitwell et al., 2018). There is growing evidence that the pathogenesis of AD is not limited to the neuronal compartment but is closely related to immune mechanisms in the brain. However, the mechanisms of neuronal death remain unclear (Whitwell et al., 2018; Dionisio-Santos et al., 2019).

In 2013, Zhang et al., observed that Nec-1 prevents neural cells from degenerating in a mouse model of AD (Qinli et al., 2013). Next, Yang SH et al. researched Nec-1, which reduces Aβ and tau abnormalities to mitigate memory loss in an AD animal model (Yang et al., 2017). Caccamo and others observed necroptosis in postmortem brains of AD patients, which was closely related to brain weight and cognitive levels. In addition, they found that RIPK1 plays a crucial role at the transcriptome level in AD. Moreover, they observed that inhibition of necroptosis reduced cell death in a mouse model of AD (Caccamo et al., 2017).

Etiologically, dysfunction of microglia plays a fundamental role in AD, and in 2017 it was found that RIPK1-dependent transcription promotes disease-associated microglia and lysosomal defects to mediate the accumulation of amyloid plaques in AD. The pseudokinase MLKL is moved from the cytosol to the plasma membrane after it is phosphorylated by the kinase RIPK3, which is required for necroptosis (Ofengeim et al., 2017). Fan et al., also found that phosphorylated MLKL was translocated from membranes through ALIX–syntenin-1–mediated exocytosis or flotillin-mediated endocytic lysosomal degradation (Fan et al., 2019). Thus, targeting RIPK1 and/or RIPK3 may provide an important therapeutic blueprint for the treatment of AD.

### Parkinson’s Disease

PD is the second most common neurodegenerative disorder. Pathologically, dopaminergic neurons in the substantia nigra (SN) pars compacta degenerate slowly and progressively (Hirsch and Hunot, 2009). The underlying causes of PD remain uncertain, but existing data support the significance of noncellular autonomic pathological mechanisms in PD, most of which are activated by glial cells and/or peripheral immune cells (Xu et al., 2017; Seo et al., 2020). This cell response to neurodegenerative changes can trigger harmful events (such as oxidative stress and cytokine receptor-mediated apoptosis), ultimately resulting in the death of dopamine cells and leading to disease progression (Liu et al., 2019; Trist et al., 2019).

Clinical studies report significant increases in dopaminergic neuron degeneration in the SN of PD patients. In contrast to the control group, RIPK1, RIPK3 and MLKL in the SN were increased at the autopsy of PD patients (Oñate et al., 2020). Downregulation of transforming growth factor *β*-activated kinase-1 (TAK1) may promote age-dependent PD in the CNS of aging humans. Mitochondrial and lysosomal dysfunction may promote intracellular RIPK1 activation and are prone to necroptosis. Inhibiting the enzyme can slow the progression of PD (Iannielli et al., 2018). Furthermore, scientists have performed some research on PC12 cells. Nec-1, which is associated with the apoptosis signaling pathway in this process, exerted a protective response against injury on dopaminergic neurons (Wu et al., 2015). In 2019, parkin was found to be an E3 ubiquitin ligase involved in PD, suggesting that parkin may alter inflammation and necroptosis by participating in ubiquitination events. The loss of parkin protected microglial cells from zVAD-induced necroptosis, thus accelerating primary neuronal injury caused by inflammation (Dionísio et al., 2019). However, further studies are needed for a detailed understanding of the mechanisms underlying these effects of necroptosis in PD.

### Amyotrophic Lateral Sclerosis

ALS is an incurable, adult-onset paralytic disease that manifests as a sporadic disease. It is caused by a decrease in motor neurons in the spinal cord, brainstem, and brain, and 10% of all ALS cases are due to genetic mutations (Chia et al., 2018). For example, mutations in the gene superoxide dismutase 1 (SOD1) lead to familial amyotrophic lateral sclerosis (fALS).

In 2014, scientists used a co-culture model system to study human sporadic astrocyte co-cultured with human embryonic stem cell-derived motor neurons, and necroptosis was found as the central feature of the death of motor neurons (Re et al., 2014). Moreover, RIPK1- and RIPK3-mediated axonal damage has been shown to occur extensively in SOD1 transgenic mice and pathological tissues from ALS patients (Saccon et al., 2013). Mutations of the optineurin (OPTN) gene promoted a marked increase in the secretion of proinflammatory cytokines, as well as neuronal cell death, in both fALS and sporadic ALS (Toth and Atkin, 2018). A lack of OPTN induces the activation of necroptosis in oligodendrocytes, leading to Wallerian-like axonal degeneration (Ito et al., 2016). It was also found that OPTN actively suppressed RIPK1-dependent signaling by regulating its turnover. In contrast to WT oligodendrocytes, OPTN^−/−^ oligodendrocytes are more susceptible to TNF-induced necroptosis, which can be inhibited by Nec-1s (Politi and Przedborski, 2016; Greco et al., 2018). Therefore, the necroptotic pathway is proposed as a novel possible target for the treatment of this incurable disease.

### Acquired Immune Deficiency Syndrome

Human immunodeficiency virus 1 (HIV-1) is a 9.7 kb retrovirus found in 1983 that was recognized as the pathogene for an increasing lethal immunodeficiency syndrome named AIDS (Selik et al., 1984). HIV enters and stays in the CNS *via* myelomonocytic cells, such as monocytes, perivascular cells, and microglia (Yadav and Collman, 2009). HIV-1 infection is described as a progressive decrease in CD4+ T lymphocytes and loss of function of the immune system. AIDS manifests in infected individuals years after the initial infection (Doitsh and Greene, 2016).

Recently, necroptosis has been biologically and pathologically researched in HIV-1-infected cells. Unlike almost all infectious diseases, HIV infection precisely targets microglia in the brain and T lymphocytes in the periphery, which are crucial components of the neuroimmune system, resulting in dysfunction of these cells. One report found that necroptosis exists in both the infected primary CD4+ T lymphocytes and CD4+ T cell lines (Pan et al., 2014). Another article suggested that caspase-8 activity was positively correlated with disease severity and programmed cell death-1 (PD-1) expression but negatively correlated with proliferation in HIV-specific CD8+ T cells (Gaiha et al., 2014). NecroX-5 could inhibit defective HIV-specific CD8+ T cell proliferation by blocking necroptosis (Kim et al., 2010; Gaiha et al., 2014). Therefore, chronic stimulation from HIV contributes to caspase-8 activity and increases the cell death of HIV-specific CD8+ T cells through the activation of necroptosis (Gaiha et al., 2014). Drug therapy combined with the inhibitor of necroptosis may improve HIV treatment.

### Retinopathy

Oxidative stress, inflammation and neurodegeneration are the main contributors in the most common retinal diseases, such as age-related macular degeneration, glaucoma and diabetic retinopathy (Bapputty et al., 2019; Dieguez et al., 2019). An unbalanced retinal immune reaction involving responses of local microglia and recruited macrophages has been specifically emphasized in retinal degenerative diseases (Akhtar-Schäfer et al., 2018).

Glaucoma is characterized by the loss of retinal ganglion cells and is a leading cause of nonreversible blindness, as well as a deteriorating neurodegenerative disease, with a probable seventy million people suffering worldwide (Tham et al., 2014). Current findings place inflammation and apoptosis as important contributors to retinal cell death under elevated pressure. RIPK1-inhibitory compound (RIC), which performs biochemical functions different from those of previous factors (Nec-1 and Compound 27; Cpd27), inhibits downstream pathways following RIPK1 activation and is mediated by necrosome composition and mitochondrial dysfunction (Do et al., 2017). Microglia play an important role in necroptosis. Necroptotic microglia produce several kinds of proinflammatory cytokines and chemokines, such as TNF-α and chemokine ligand 2 (Chen et al., 2019a)^,^ (Qin et al., 2018). RIPK1- and RIPK3-dependent necroptosis existed in microglia of mouse models with degenerative retina or acute retinal neural injury (Huang et al., 2018b). Necrostatin-1 blocked necroptosis, depressing microglia-mediated inflammation, which protected retinal degeneration or reduced neural injury *in vivo*. In the pathway, knockdown of TLR4 reduces RIPK1 and RIPK3 expression to suppress microglial necroptosis and depress retinal inflammation, which suggests that TLR4 signaling participates in necroptosis-mediated microglial inflammation (Huang et al., 2018b). Thus, microglia in the retina provoke inflammatory activation through TLR4-mediated necroptosis, which aggravates retinal neural damage and retinal degeneration.

In regard to ocular trauma, blast-exposed patients experience subsequent vision loss even after a healthy ophthalmological exam. Increased intraocular RIPK3 suggests that the photoreceptors are depleted because of necroptosis. TNF-a and RIPK3 exacerbate the activation of microglia, indicating that RIPK3 may also result in oxidative stress in the outer retina and lead to progressive cell loss (Thomas et al., 2019). Inhibitors of necroptosis are thought to be a new promising strategy to promote neuroaxonal survival and remyelination, potentially preventing disability in retinal diseases.

## Necroptosis Inhibitors

Several specific necroptosis inhibitors aimed at RIPK1, RIPK3, or MLKL have been found or developed, such as Nec-1, Nec-1s, cpd27, and GSK872. Studies of these specific necroptosis inhibitors have shown therapeutic effects in various neuroinflammatory diseases. In animal models of TBI, Nec-1 (a kind of RIPK1 inhibitor) inhibits apoptosis and autophagy (Wang et al., 2012). Nec-1 also reduces Aβ and tau abnormalities in an AD animal model (Yang et al., 2017). Dabrafenib (an inhibitor of RIPK3 kinase-dependent necroptosis) reduces ischemic brain damage in mice (Cruz et al., 2018). The novel MLKL inhibitors have been proven to be promising tools for studying the biological function of MLKL and as druggable targets of necroptosis. However, the MLKL inhibitors currently known are few (such as TC13172, necrosulfonamide and GW806742X), and no MLKL inhibitor has reached the clinical trial stage (Chen et al., 2017; Yan et al., 2017). However, most studies have yet to conduct clinical trials, and there are still questions about human safety (Chen et al., 2019b).

## Conclusion

Necroptosis is a new type of programmed necrosis that can be activated by multiple kinds of extracellular and intracellular stimuli. Our understanding of the underlying molecular mechanism and biological function of necroptosis has increased in recent years. In general, necroptosis achieves physiological and pathological effects through the TNF or TLR pathway. Depending on the inhibition of caspase-8 activation, RIPK1, RIPK3, MLKL, FADD and procaspase-8 form complex IIb, which leads to necroptosis. The mechanism by which necroptosis is achieved through the TLR3 and TLR4 pathways is unclear. In the rat model of hydraulic shock brain injury, RIPK1 and 3, MLKL and proinflammatory factors were increased in the cortex (Liu et al., 2016). RIPK1 and 3 and the NLRP3 inflammasome were reported to participate in necroptosis of ICH (Shen et al., 2017). In chronic neuroinflammatory diseases such as AD, RIPK3 impacts necroptosis by phosphorylating MLKL (Ofengeim et al., 2017; Fan et al., 2019). Although the role of necroptosis in inflammatory or apoptotic pathologies has been appreciated, our knowledge of the involvement and impact of necroptosis in neuroinflammatory diseases remains limited. Hence, a deeper understanding of necroptosis in neuroinflammatory diseases, such as AD and stroke, could be beneficial for providing insights into the mechanisms of neuronal death and clinical treatments. The precise mechanism of plasma membrane breakage induced by MLKL in necroptosis remains to be uncovered. Whether MLKL plays a role in necroptosis as a carrier protein of some particular proteins, from cytoplasm to nuclei, also needs further study. While some small-molecule inhibitors of RIPK1 and RIPK3 have made some progress in clinical trials, the efficacy of treatment remains to be confirmed by multicenter experiments.

To conclude, we highlighted the increasing evidence about the role of necroptosis in various neuroinflammatory diseases. In the future, improvement of the application of such signaling inhibitors may remove obstacles for replacement therapies for neurological diseases.

## Glossary

Aβ: Amyloid-β
AD: Alzheimer’s disease
AIDS: Acquired Immune Deficiency Syndrome
ALS: Amyotrophic lateral sclerosis
c-FLIP: cellular FADD-like interleukin-1β converting enzyme inhibitory protein
cIAP1: Cellular inhibitor of apoptosis protein 1
CNS: Central nervous system
DRs: Activated death receptors
FADD: Fas-associated death domain
fALS: Familial amyotrophic lateral sclerosis
FPI: Fluid percussion injury
HIV-1: Human immunodeficiency virus 1
ICH: Intracerebral hemorrhage
IKK: IκB kinase
IRI: Ischemia-reperfusion injury
JE: Japanese encephalitis
JEV: Japanese encephalitis virus
LPS: lipopolysaccharide
MLKL: Mixed lineage kinase domain-like protein
Nec-1: Necrostatin-1
NEMO: Nuclear factor-Kappa B essential modulator
NLRP3: NLR Family Pyrin Domain Containing 3
OPTN: optineurin
PD: Parkinson’s disease
PD-1: programmed cell death-1
RIC: RIPK1-inhibitory compound
RIPK1: Receptor-interacting protein kinase 1
RIPK3: Receptor-interacting protein kinase 3
sALS: sporadic amyotrophic lateral sclerosis
SN: Substantia nigra
SOD1: Superoxide dismutase 1
TAK1: Transforming growth factor β-activated kinase-1
TBI: Traumatic brain injury
TICAM-1: TIR domain-containing adaptor molecule 1
TLR: Toll-like receptor
TNF: Tumor necrosis factor
TNFR1: TNF receptor 1
TRADD: TNFR-associated death domain
TRIF: Toll/IL-1 receptor domain-containing adaptor inducing IFN-β
WNV: West Nile virus
WT: wild-type
